# Potential Use of Cefiderocol and Nanosilver in Wound Dressings to Control Multidrug-Resistant Gram-Negative Bacteria

**DOI:** 10.3390/molecules30153072

**Published:** 2025-07-23

**Authors:** Żaneta Binert-Kusztal, Agata Krakowska, Iwona Skiba-Kurek, Magdalena Luty-Błocho, Anna Kula, Aldona Olechowska-Jarząb, Przemysław Dorożyński, Tomasz Skalski

**Affiliations:** 1 Department of Inorganic Chemistry and Pharmaceutical Analytics, Faculty of Pharmacy, Jagiellonian University Medical College, Medyczna 9 Street, 30-688 Krakow, Poland; zaneta.binert@uj.edu.pl (Ż.B.-K.); przemyslaw.dorozynski@uj.edu.pl (P.D.); 2 AGH University of Krakow, Department of Analytical Chemistry and Biochemistry, Faculty of Materials Science and Ceramics, Al. Mickiewicza 30, 30-059 Krakow, Poland; 3 Department of Pharmaceutical Microbiology, Faculty of Pharmacy, Jagiellonian University Medical College, Medyczna 9 Street, 30-688 Krakow, Poland; iwona.skiba@uj.edu.pl; 4 Department of Microbiology, University Hospital, Marii Orwid 11 Street, 30-680 Krakow, Poland; aolechowska@su.krakow.pl; 5 AGH University of Krakow, Faculty of Non-Ferrous Metals, al. Mickiewicza 30, 30-059 Krakow, Poland; mlb@agh.edu.pl (M.L.-B.); kula@agh.edu.pl (A.K.); 6 Biotechnology Centre, Silesian University of Technology, Krzywoustego 8 Street, 44-100 Gliwice, Poland

**Keywords:** cefiderocol, synthesis, synergy, AgNPs, Gram-negative bacteria, wounds, bacterial infections

## Abstract

This study evaluated the antimicrobial efficacy of cefiderocol and various forms of silver (ionic and nanoparticulate) as potential components of wound-dressing reagents against both reference and multidrug-resistant (MDR) Gram-negative bacteria. The anticipated synergistic effect between cefiderocol and nanosilver was not consistently observed; in fact, for reference strains, the combination was less effective than cefiderocol alone. However, in MDR and cefiderocol-resistant *A. baumannii* strains, combining both agents enhanced antibacterial efficacy. Notably, the effectiveness of silver did not increase with concentration, and low or medium nanosilver concentrations were often more effective. Mechanistically, high concentrations of silver may antagonize cefiderocol’s action by inhibiting bacterial surface proteins involved in siderophore-mediated uptake. Generalized linear modeling confirmed that the strain type, silver form, concentration, and their interactions significantly influenced inhibition zones. These findings highlight the importance of agent selection, concentration, and formulation in designing effective antimicrobial wound dressings. They also suggest that further research is needed to optimize such combination therapies for clinical use.

## 1. Introduction

Non-healing wounds, often referred to as chronic wounds, can be a significant medical issue and may arise from various underlying conditions [1,2]. Chronic wounds are often more susceptible to infections, which can complicate the healing process and lead to systemic issues [3]. The eradication of bacteria from the wound bed is a fundamental and most difficult problem in initiating the healing process during wound management [4]. A prolonged process of inflammation and chronic infection can complicate healing and lead to systemic issues and impaired wound healing. Many chronic wounds harbor biofilms, which are communities of bacteria encased in a protective matrix. Biofilms make it difficult for antibiotics and the immune system to eradicate the bacteria, allowing the infection to persist and contribute to ongoing inflammation [5,6]. Infections in wounds can prevent healing by causing tissue damage, increasing inflammation, and potentially leading to systemic complications. Because of prolonged antimicrobial therapies, many bacterial strains present in non-healing wounds may be antibiotic-resistant [7,8]. This resistance can make it challenging to eradicate infections using standard therapies, requiring more aggressive treatments or alternative antibiotics [9,10]. Hence, it is fully justified to undertake research aimed at developing new solutions, such as the use of effective antibiotics for external wounds or their combination with nanoparticles with scientifically proven antibacterial effects [11,12,13,14].

A promising solution proposed in this research paper is cefiderocol, a fourth-generation cephalosporin antibiotic that has shown positive results in the treatment of infections caused by a broad spectrum of multidrug-resistant (MDR) Gram-negative bacteria [15,16]. It was designed to mimic the structure of siderophores, molecules that bacteria use to capture iron from their environment. By mimicking siderophores, cefiderocol may use bacterial iron transport systems to enter cells. This is particularly important because many Gram-negative bacteria require iron for growth and may regulate iron uptake mechanisms [17,18,19,20]. The third-phase clinical trials for APEKS-cUTI and CERDIBLE-CR [21,22] confirmed good results for the internal application of antibiotic therapy in patients (especially for nosocomial pneumonia therapy). This broad spectrum makes it a valuable option for treating infections in non-healing wounds that may not respond to other antibiotics. Currently, there are no reports in the literature regarding the use of cefiderocol in the treatment of difficult-to-heal wounds [23].

Cefiderocol was designed to avoid certain mechanisms of bacterial resistance, such as the production of extended-spectrum beta-lactamases (ESBLs) and carbapenemases, which are common among resistant Gram-negative bacteria [24,25]. This ability to withstand degradation by these enzymes makes it a powerful option for fighting difficult infections. Cefiderocol is bactericidal, meaning that it not only inhibits bacterial growth, but also kills bacteria [26]. This is especially important in acute cases where rapid eradication of infection is needed for wound healing [27]. Clinical studies have shown that cefiderocol can be effective in treating complicated urinary tract infections and infections associated with MDR pathogens, suggesting its potential use in wound infections as well. While biofilms pose a significant challenge in treating chronic wounds, cefiderocol’s ability to penetrate and act against bacteria in biofilms may also enhance its efficacy in such cases. Cefiderocol has a unique structure that allows it to effectively cross the outer membrane of Gram-negative bacteria, which is key to its antimicrobial activity.

The efficacy of cefiderocol depends in part on the availability of iron in the bacterial environment. Interaction with iron via a siderophore-like mechanism is crucial for its uptake by bacterial cells. The presence of other metal ions in the environment may affect the overall stability, solubility, or competitive binding of cefiderocol under various conditions. The availability of Fe^3+^ is crucial for cefiderocol uptake and activity. The changes in pH or environment that alter Fe^3+^ availability (e.g., conversion to Fe^2+^ under acidic or anaerobic conditions) can reduce cefiderocol efficacy by limiting complex formation [28,29]. Much more data on ion interaction with cephalosporins are obtained for cefadroxil, with zinc reducing free drug availability through stronger binding affinity and transepithelial drug transport inhibition [30]. Cephalosporin interaction with toxic ions such as bismuth and gallium may increase antibacterial efficacy, suggesting that this is a promising approach to the multidrug resistance crisis [31]. The combination of cefiderocol with other metal ions, commonly used in non-healing wound treatment such as nanosilver has not been widely studied, in spite of a potential synergy with other cephalosporins against *P. aeruginosa* and *E. coli* [32].

Silver nanoparticles (AgNPs) exhibit strong antimicrobial activity against a wide range of pathogens, including multidrug-resistant (MDR) Gram-negative and Gram-positive bacteria, as well as fungi. AgNPs demonstrate multiple concurrent mechanisms of action, including membrane damage. AgNPs adhere to bacterial cell membranes through electrostatic interactions, causing structural damage, depolarization, and increased permeability [33,34], DNA interaction and enzyme inactivation, which are more effective and broader in spectrum compared to gold nanoparticles, metal oxide nanoparticles, or polymeric nanoparticles [35,36,37,38,39]. AgNPs also show low cytotoxicity to mammalian cells at concentrations effective against bacteria, whereas some metal oxide nanoparticles (e.g., ZnO or TiO_2_) may exhibit higher cytotoxicity or induce oxidative stress in host tissues [40]. AgNPs not only control infection, but also support wound healing by modulating inflammation, stimulating fibroblast migration, and aiding collagen deposition. These studies highlight the well-documented, multifaceted role of silver nanoparticles in wound healing, extending beyond infection control, with more consistent and robust supporting evidence.

AgNPs, in combination with cephalosporins, significantly reduce biofilm formation by up to 60% [41,42]. AgNPs conjugated with antibiotics (e.g., amikacin) exhibited tenfold higher efficacy against resistant strains compared to the antibiotics alone [43]. A similar synergy could enhance the activity of cefiderocol against multidrug-resistant Gram-negative bacteria.

While direct studies on cefiderocol-AgNPs combinations are lacking, existing evidence suggests that nanosilver could potentiate cefiderocol’s activity through membrane disruption, biofilm inhibition, and ROS generation. However, competitive metal binding and resistance risks warrant further investigation. This synergy may offer a promising strategy for combating severe infections caused by biofilm-forming, multidrug-resistant pathogens. The goal of this paper was to test the potential application of cefiderocol with nanosilver particles to increase the efficacy against drug resistance clinical strains of *Acinetobacter baumannii* and reference strains. We hypothesize that increased membrane disruption could improve drug penetration and better control of strains. However, there is a risk that AgNPs at high concentrations may interfere with iron transport proteins (e.g., TonB, ExbB-ExbD), negatively influencing energy-dependent cefiderocol uptake.

## 2. Results and Discussion

**Figure 1:**
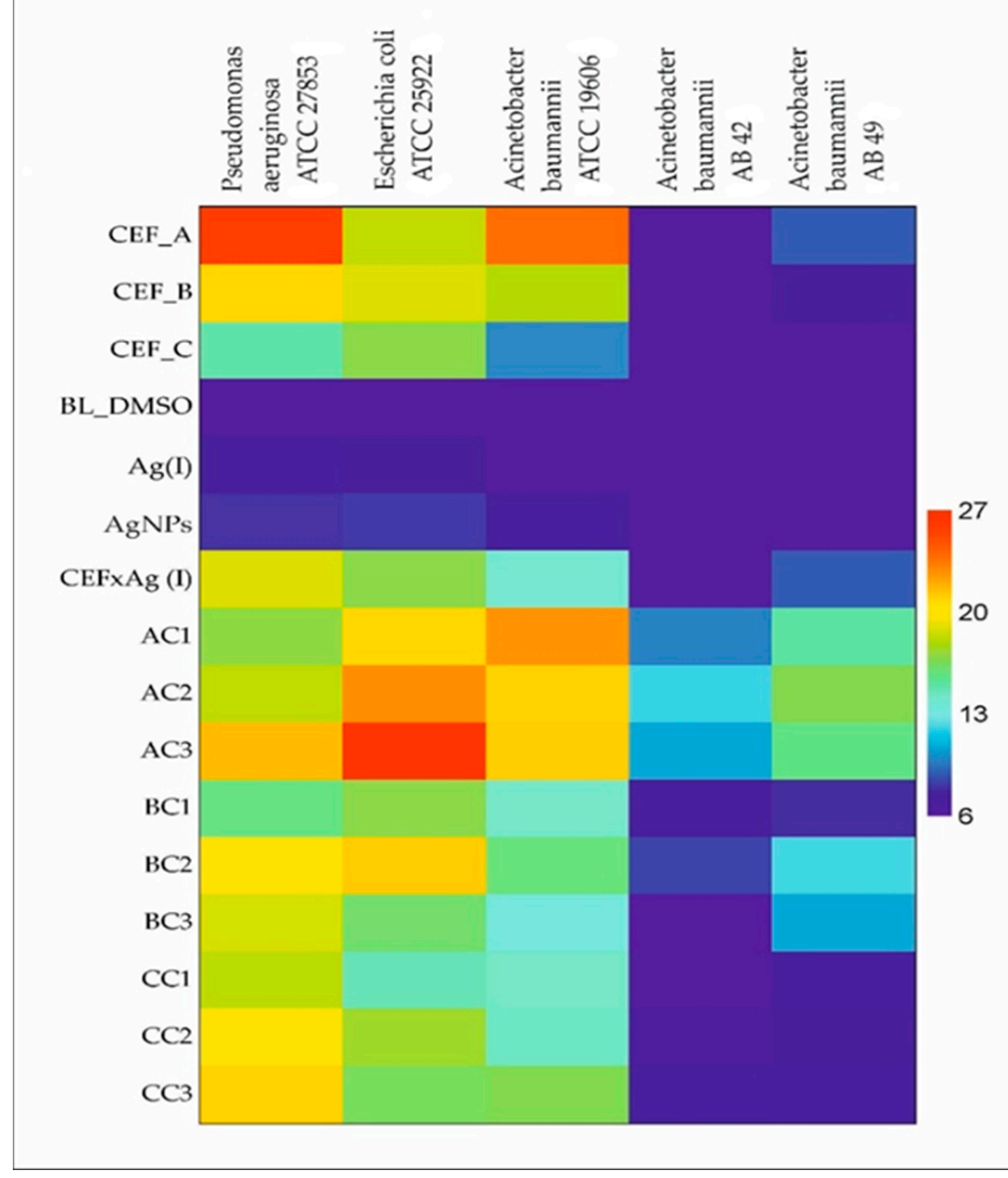
Heat map of mean inhibition area of selected antimicrobial agents used externally on bacterial strains using the disk diffusion method. Notation: CEF—cefiderocol, CEF_A—CEF at initial concentration 1.5 mg/mL; CEF_B—CEF at initial concentration 0.15 mg/mL; CEF_C—CEF at initial concentration 0.015 mg/mL, BL—blank test, DMSO—dimethyl sulfoxide, Ag(I)—silver ions, AgNPs—silver nanoparticles, AC1—CEF concentration 0.75 mg/mL + AgNPs (1 mM), AC2—CEF concentration 0.75 mg/mL + AgNPs (0.5 mM), AC3—CEF concentration 0.75 mg/mL + AgNPs (0.25 mM), BC1—CEF concentration 0.075 mg/mL + AgNPs (1 mM), BC2—CEF concentration 0.075 mg/mL + AgNPs (0.5 mM), BC3—CEF concentration 0.075 mg/mL + AgNPs (0.25 mM), CC1—CEF concentration 0.0075 mg/mL + AgNPs (1 mM), CC2—CEF concentration 0.0075 mg/mL + AgNPs (0.5 mM), CC3—CEF concentration 0.0075 mg/mL + AgNPs (0.25 mM)).

The efficacy of particular components of potential wound dressing reagents in solid form is presented in Figure 1. Additionally, a control sample containing only DMSO (denoted as BL_DMSO, Figure 1) was tested to assess its intrinsic antibacterial activity.

Among analyzed strains, the reference *P. aeruginosa*, *E. coli* and *A. baumannii* were susceptible to cefiderocol and showed dose-dependent effects. Both clinical MDR strains of *A. baumannii* were not sensitive to cefiderocol, nor to various silver forms. A synergistic effect of both antimicrobial agents (antibiotic and nanosilver) is not evident. In the case of reference strains, combined effect of cefiderocol and nanosilver was much less effective than cefiderocol alone, but MDR and cefiderocol-resistant strains of *A. baumannii* were more successful when both agents were applied together.

To assess antibacterial activity, we also examined the individual effects of Ag(I) ions and pure AgNPs on the following strains: *Pseudomonas aeruginosa* PA ATCC 27853, *Acinetobacter baumannii* AB ATCC 19606, *Escherichia coli* EC ATCC 25922, and two clinical isolates of *A. baumannii* AB 42 and AB 49. The results presented in Figure 1 suggest that AgNPs exhibit slightly higher antibacterial activity compared to Ag(I) ions. Nonetheless, comparative tests evaluating potential synergistic effects between cefiderocol and Ag(I) (CEF × Ag(I)), as well as with AgNPs (AC1), were conducted (see Figure 1). The findings demonstrated that AgNPs were generally more active than Ag(I) ions, except in the case of *P. aeruginosa* ATCC 27853, thereby supporting their broader antibacterial potential across multiple strains.

Probably, the enhanced antibacterial properties of silver nanoparticles result from their physicochemical properties (shape, size, surface activity), which support drugs by improving solubility, degradation, and combination therapy [23]. The small size of nanoparticles guarantees their high activity, which was also confirmed in other application studies [4]. Surprisingly, samples containing a constant amount of cefiderocol and different concentration of AgNPs (samples AC1–AC3, Figure 1) have different antibacterial activity. Comparing the samples containing highest and smallest amount of AgNPs (samples AC1 and AC3, respectively) showed that antibacterial activity against the selected bacteria increased as the amount of metal decreased.

The relationship between the silver form (ionic or nanoparticulate silver) at a 0.0075 mg/mL concentration of cefiderocol was analyzed using generalized linear mixed modeling with a gamma distribution of the data. All combinations (strain, form, and concentration significantly influenced the inhibition zone diameter (Table 1).

**Figure 2:**
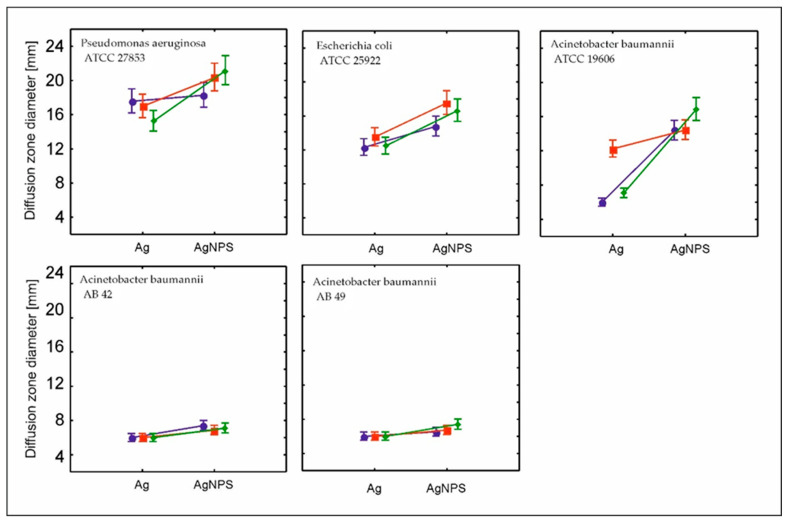
Mean ± confidence interval of the diameter of the bacterial growth inhibition zone in relation to the applied concentration of silver and silver nanoparticles (navy blue—1 mM, red—0.5 mM, green—0.25 mM). (PA ATCC 27853—*Pseudomonas aeruginosa*, AB ATCC 19606—*Acinetobacter baumannii*, EC ATCC 25922—*Escherichia coli*, AB 42—*Acinetobacter baumannii* (clinical strain), AB 49—*Acinetobacter baumannii* (clinical strain)).

The clinical strains were much less sensitive to nanosilver form and concentration than standard strains; however, the strain control was visible (Figure 2).

It was also remarkable that the zone inhibition diameter was not related to the silver concentrations. We obtained much better results for strain eradication with medium or the smallest amounts of both nanosilver forms. The nanoform of silver seems however to be more efficient than ionic form with respect tocefiderocol concentration at level 0.0075 mg/mL. Nanosilver releases silver ions (Ag^+^) gradually due to its structural stability, whereas ionic silver releases all Ag^+^ immediately upon contact [43]. This sustained release ensures prolonged antimicrobial activity without overwhelming the wound environment, which could disrupt cefiderocol’s targeted action. The plant-based capping agents in nanosilver also prevent aggregation, maintaining its efficacy. We should also remember that cefiderocol relies on bacterial iron-uptake systems for transport [44]. Ionic silver’s rapid diffusion might impair cefiderocol’s “Trojan horse” mechanism [42] of siderophore transport through an external membrane. It may also explain why the zone inhibition for bacterial strains is dose-dependent; however, the efficiency is much better at lower concentrations of silver. Cefiderocol—a paracephalosporin conjugated with siderophore—is actively transported through the outer membrane of Gram-negative bacteria. The enzymatic system of bacteria recognizes the antibiotics as a source of iron. The Fe^3+^-cefiderocol complex is actively imported through species-specific outer membrane transporters which are strain-dependent. *Pseudomonas aeruginosa* primarily uses PiuA receptors [45,46]; meanwhile, *E. coli* relies on CirA and Fiu transporters [46]. This active transport is energy-dependent, utilizing the TonB-ExbB-ExbD protein complex to power translocation across the membrane [47].

Silver inhibits the activity of bacterial surface proteins. High concentrations of silver act antagonistically to the mechanism of action of the Trojan horse antibiotic, and consequently, the effectiveness of the antibiotic will decrease. By inhibiting the protein that recognizes the siderophore, the activity of the antibiotic also declines. A similar mechanism can be observed in strains resistant to the action of cefiderocol. Protein mutations in surface proteins slowed down the process of siderophore uptake, simultaneously preferring mutants in therapy with this antibiotic.

**Figure 3:**
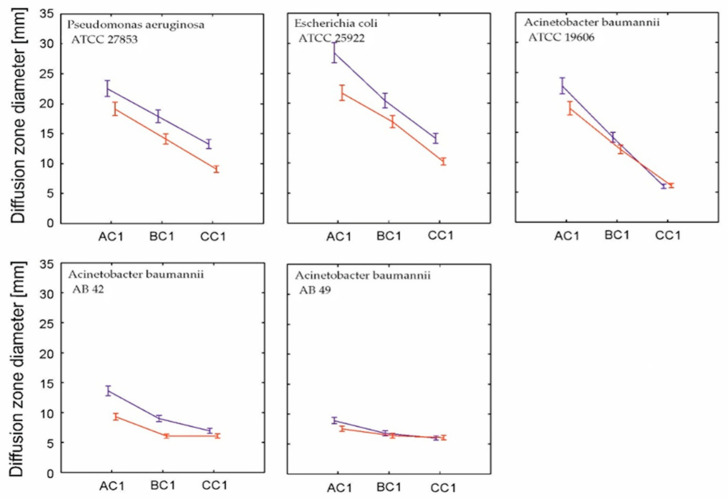
The reletionship between the mean diameter of the inhibition zone (±confidence interval) and vertical layer orientation of cefiderocol and nanosilver. (navy bluecefiderocol in direct touch to strains, red—nanosilver in direct contact with bacterial strains. AC1—cefiderocol concentration 0.75 mg/mL + AgNPs (1 mM), BC1—cefiderocol concentration 0.075 mg/mL + AgNPs (1 mM), and CC1—cefiderocol concentration 0.0075 mg/mL + AgNPs (1 mM)).

We also wanted to identify if the surface layer location between silver and cefiderocol is important for its application. We tested the influence of the position of AgNPs in relation to cefidercol in vertical layer organization (Figure 3). A generalized linear mixed model showed that both vertical zonation and cefiderocol concentration significantly determined the diffusion zone diameter, and this relationship was strain-dependent (Table 2).

The effectiveness of strain control was higher when cefiderocol was directly in contact with bacterial strains and their biofilm. Cefiderocol is a siderophore cephalosporin antibiotic that exploits bacterial iron uptake systems to actively penetrate Gram-negative bacteria [48,49]. Nanosilver acts mainly through the release of silver ions (Ag^+^), which disrupt bacterial membranes and proteins, including those responsible for cefiderocol transport. Positioning nanosilver in the upper layer allows the controlled release of silver ions into the wound environment [50], providing sustained antimicrobial action without interfering with cefiderocol’s direct bacterial targeting.

**Figure 4:**
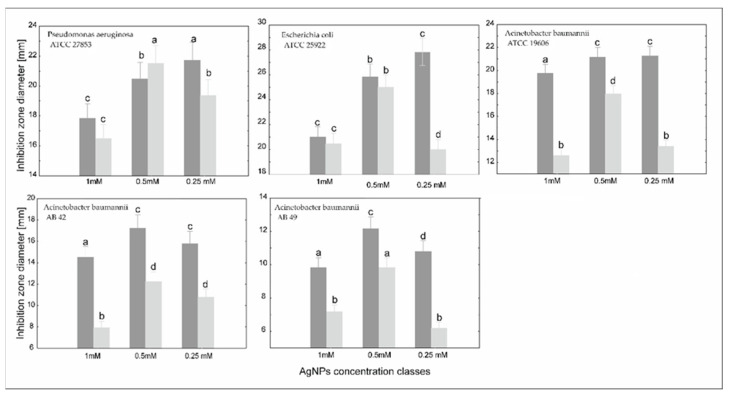
The combined effect of cefiderocol concentration (0.75 µM—darker and 0.075 µM—lighter) and nanosilver concentrations on the mean ± confidence interval of inhibition zone diameter of standard (PA ATCC 27853, EC ATCC 25992, AB ATCC 19606) and clinical (AB 42, AB 49) multidrug-resistant strains revealed from generalized linear modeling with a gamma distribution. Means that share the same letter are not statistically different from each other according to the Tukey pairwise test.

We observed the synergistic effect of cefiderocol concentration and nanosilver concentration in relation to clinical and reference strains (Figure 4).

**Figure 5:**
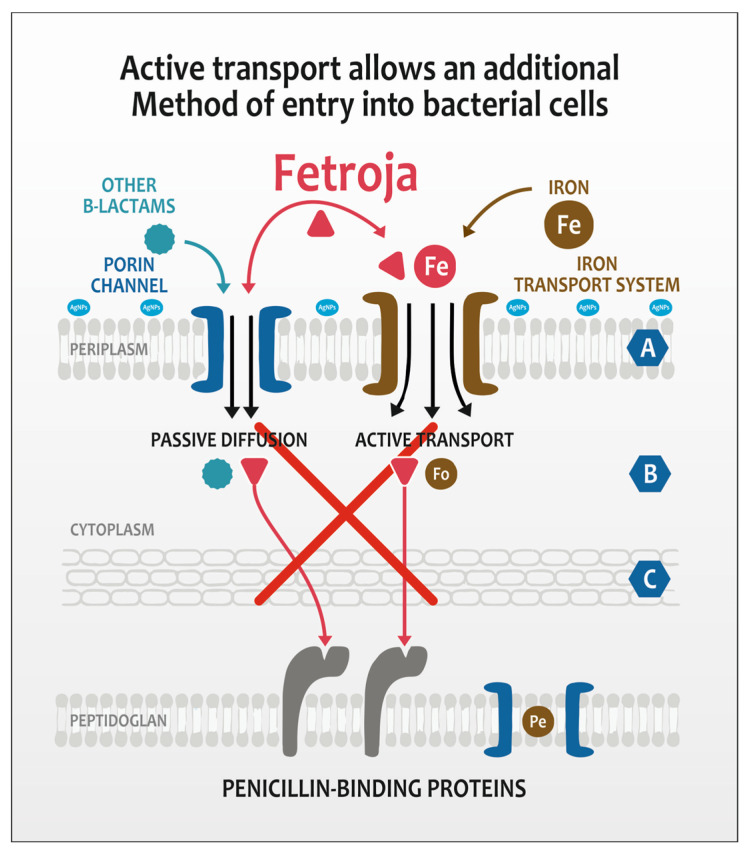
Mechanism of iron transport into the bacterial cell and the potential impact of nanosilver.

To obtain the optimal synergy between cefiderocol concentration and nanosilver amount, generalized mixed modeling was applied (Table 3). The combination of two antimicrobial agents indicated the significant effect on the inhibition zone area. However, as expected, it also depended on the strain. Generally, the higher the cefiderocol concentration, the larger the inhibition zone in every nanosilver concentration class (Figure 4). The only exception is *P. aeruginosa* at a medium nanosilver concentration. In this case, silver toxicity is more influential. *Acinetobacter* MDR clinical strains showed inhibition in all groups of antibacterial agents, in spite of showing no inhibition to pure silver and cefiderocol. It was, however, remarkable that efficacy was higher when the concentration of cefiderocol was higher and concentrations of nanosilver lower. Cefiderocol, a siderophore cephalosporin, could interact negatively with higher concentrations of nanosilver (AgNPs) due to mechanisms that disrupt its iron-dependent uptake, alter its stability, or even both. Zink ions strongly interact with cephalosporins similar to cefiderocol, forming complexes that reduce free drug availability (Figure 5).

Cefiderocol (Fetroja^®^) is a siderophore cephalosporin that exploits bacterial iron uptake pathways to traverse the outer membrane of Gram-negative bacteria [51]. Its entry is facilitated by specific bacterial proteins involved in siderophore-mediated iron transport. Our studies have demonstrated that high concentrations of silver ions or silver nanoparticles can disrupt these iron uptake mechanisms, thereby antagonizing cefiderocol’s antibacterial efficacy. Three key pathways appear susceptible to interference by nanosilver: TonB-dependent transporters, outer membrane siderophore receptors, and general outer membrane porins. The TonB-ExbB-ExbD protein complex energizes the active transport of siderophore-antibiotic complexes, such as cefiderocol-Fe^3+^, through outer membrane receptors [52]. Nanosilver can bind to thiol groups or induce conformational changes in these proteins, impairing the energy transduction necessary for cefiderocol entry. The second group includes siderophore receptors such as CirA, Fiu, and FepA, which recognize and transport siderophore–iron complexes (and cefiderocol–iron mimics) into the periplasm; nanosilver exposure can cause structural alterations or protein aggregation in these receptors [29]. Finally, cefiderocol may also enter via alternative pathways involving general porins such as OmpC, OmpF, and OmpA. Silver nanoparticles have been shown to modify the structure and function of these porins, reducing membrane permeability and further limiting cefiderocol access to the periplasm [53]. Further experimental studies are necessary to fully elucidate these mechanisms, particularly in cefiderocol-resistant bacterial strains and mutants.

For cefadroxil, a related cephalosporin, Zn^2+^ showed the strongest binding affinity and significantly inhibited transepithelial drug transport by forming stable complexes [54,55]. Copper ions also form complexes with cephalosporins, with a binding affinity slightly lower than zinc but still significant. Cu^2+^ can inhibit drug uptake by complex formation [54]. Also, cobalt ions exhibit moderate binding affinity to cephalosporins, less than Zn^2+^ or Cu^2+^ but more than Fe^3+^ and Al^3+^. It is possible, then, that lower-oxidation-rate metal ions compete with Fe^+3^ for siderophore binding. Siderophores are evolutionarily optimized to bind Fe^3+^ due to its specific ionic radius (~0.65 Å) and preference for octahedral coordination. Metal ions with similar radii and coordination preferences (e.g., Ga^3+^ at ~0.62 Å) can effectively compete with Fe^3+^ for binding [56,57]. Silver nanoparticles (AgNPs) do not have a specific “ionic radius” because they are composed of many silver atoms clustered together, typically ranging from 1 to 100 nanometers (nm) in overall particle diameter [58].

A more reasonable explanation is related to antimicrobial properties of nanosilver particles, especially their ability to bind to proteins and enzymes, interfering with metabolic processes [59]. AgNPs can bind to and inactivate membrane-bound enzymes and siderophore receptors by interacting with thiol groups and other electron-donating atoms (oxygen, nitrogen, phosphorus), potentially blocking or altering the function of these transport proteins (TonB-dependent transporters (TBDTs) [60]. There is however problem with the mechanism of its binding. TBDTs have a 22-stranded β-barrel with an N-terminal plug domain and a conserved TonB box motif [61]. The key siderophore transport function of TBDTs does not specifically depend on free thiol (–SH) groups which interaction is characteristic to AgNPs binding. This suggests that silver nanoparticles’ binding to thiol groups likely affects other membrane proteins or enzymes rather than directly targeting the TBDTs’ siderophore-binding sites. Studies show that AgNPs induce changes in the abundance and function of OMPs such as OmpC, OmpF, and OmpA in *Escherichia coli* and other Gram-negative bacteria [62]. These proteins contain cysteine residues with thiol groups that can bind silver ions, leading to protein dysfunction. OmpC and OmpF are porins involved in passive diffusion of small molecules, and their impairment disrupts membrane permeability and nutrient transport [46]. Cefiderocol then may still passively diffuse through porins to some extent, especially in MDR clinical strains resistant to cefiderocol, but this might to be an alternative route of entry and becomes critical for its antibacterial efficacy.

## 3. Experimental Section

### 3.1. Materials Preparation

#### 3.1.1. Preparation of Unmodified Silver(I) Solutions

A fresh 2 mM AgNO_3_ solution was used as the metal precursor. To prepare this solution, a specific amount of powder (approximately 3.4 mg) was dissolved in 10 mL of DMSO. This compound was ready for use in AgNPs synthesis (see Section 3.1.2). In experiments examining the effect of silver ions on bacterial performance, the Ag(I)-containing solution was mixed with DMSO at a 1:1 volumetric ratio. Initially, this mixture was adjusted to a 1 mM concentration, which is comparable to the concentration of the silver nanoparticles used.

#### 3.1.2. Synthesis of AgNPs

Silver nanoparticles were produced using a chemical reduction method. In this method, a solution containing 2 mM AgNO_3_ was mixed with a solution of ascorbic acid (20 mM) as a reducing agent in a glass vessel (volumetric ratio 1:1). All reagents were freshly prepared before AgNPs synthesis. However, before mixing, both reagents were kept at 28–30 °C in a thermostatic bath for 10 min. After this time, the reagents were mixed. Next, the colorless solution (containing silver ions) turned light yellow (t = 2 min) and then yellow (t = 10 min). The color of the solution, the positive Tyndall effect, and a registered LSPR (localized surface plasmon resonance) maximum at about 420 nm confirm the presence of silver nanoparticles. For further studies, selected samples containing AgNPs were mixed with cefiderocol solution in a 1:1 volumetric ratio. Then, the prepared mixture (10 µL) was dispersed on filter paper. All samples were protected from light and heat.

#### 3.1.3. Preparation of Cefiderocol (Fetroja^®^) Solutions

The analytical-grade medicine (S-649266) was produced by Shionogi Incorporations (Osaka, Japan). Cefiderocol (S-649266) was the main active substance. It contained 1000 mg of cefiderocol sulfate tosylate drug product containing sucrose (900 mg per vial), sodium chloride (216 mg per vial), and sodium hydroxide (in sufficient quantity).

The test solutions of silver ions (A), silver nanoparticles (AgNPs) (B), and cefiderocol (C) used in the experiment were prepared according to Table 4.

### 3.2. Method Analysis

#### 3.2.1. Microbiological Analysis

The examination of silver nanoparticles and their combinations with cefiderocol was performed using the disk diffusion method of Kirby and Bauer on Mueller-Hinton Agar (bioMérieux, Warsaw Poland). The reference strains used for quality control and antimicrobial susceptibility testing were: *Escherichia coli* ATCC 25922, *Pseudomonas aeruginosa* ATCC 27853 and *Acinetobacter baumannii* ATCC 19606 as well as multidrug-resistant clinical strains, including those resistant to cefiderocol, namely *Acinetobacter baumannii* (AB 42), *Acinetobacter baumannii* (AB 49). The research obtained the necessary consent of the Research Ethics Committee of the Collegium Medicum of the Jagiellonian University in Krakow dated 29 November 2024, no. 118.0043.1.391.2024. The reference and clinical strains were suspended in 0.85% saline solution (bioMérieux, Warsaw, Poland), plated on the culture medium, and then the diffusion disks were placed. Samples containing pure silver (Ag(I) ions), nanosilver, cefiderocol, and cefiderocol mixed with silver nanoparticles were tested. The plates were incubated at 37 °C for 24 h. After this time, the inhibition zones were measured. The results were interpreted according to the European Committee on Antimicrobial Susceptibility Testing (EUCAST) criteria (breakpoint table ver. 14.0, 2024) [63].

The solutions of silver nanoparticles (B) in combination with cefiderocol (C) for microbiological analysis were prepared according to Table 5.

#### 3.2.2. UV-Vis Analysis

For UV-Vis spectrophotometry, silver nanoparticles were analyzed using spectrophotometry (Shimadzu 2501, Kyoto, Japan) within the wavelength range of 190–900 nm, owing to their distinctive optical properties. Moreover, the reagents used for AgNPs synthesis, i.e., solutions containing silver ions and ascorbic acid as well as cefiderocol for nanoparticles modification, were also analyzed. During analysis, DMSO was used as a reference solution in all cases. Experiments were performed in a quartz cuvette (optical path 1 cm) and in a thermostatic measurement cell.

**Figure 6:**
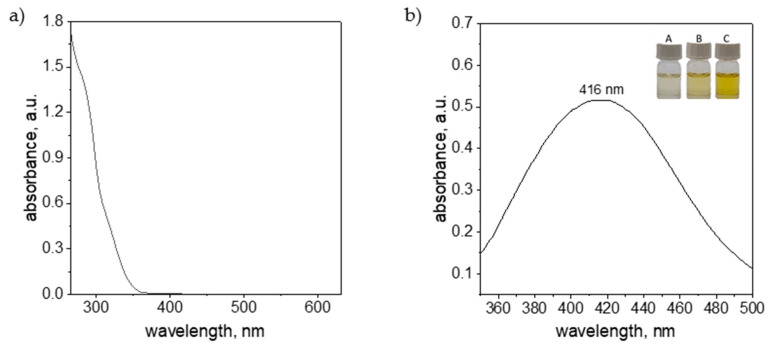
UV-Vis spectrum of solution containing cefiderocol (**a**) and AgNPs (**b**) together with the color of the sample evolution (Sample A—1 min.; B—5 min. and C—10 min.). Conditions: 2 mM Ag(I), 20 mM AA, DMSO was used as solvent, T = 20 °C.

In the case of ascorbic acid, as well as silver ions dissolved in DMSO solution, it was not possible to collect UV-Vis spectra due to strong signal coming from the solvent (appearance of strong absorption signal below 260 nm). However, it was possible to register spectrum for solution containing cefiderocol and nanoparticles. The results obtained are shown in Figure 6a.

The registered spectrum for cefiderocol did not have characteristic peak(s) in considered wavelength range (Figure 6a). A pronounced increase in spectral intensity was observed below 350 nm. Data acquisition below 250 nm was not feasible due to instrument detection limits.

The silver nanoparticles obtained in the experiments were formed as a result of the reduction reaction of silver ions using ascorbic acid. As a result, the colorless solution Ag(I) ions changed color to light yellow (samples A, B, Figure 6b) and yellow (sample C, Figure 6b). The occurrence of the yellow color of the solution and the positive Tyndall effect suggest the presence of a metallic phase. The obtained solution was analyzed using spectrophotometry and the registered spectrum was shown in Figure 6b. The colloidal silver with intense yellow color has characteristic spectrum with maximum at 416 nm, and this is consistent with the data in the literature [44]. The location of the maximum suggests the spherical shape of the nanoparticles and their size between 20 and 40 nm (particle size confirmed by microscopic examination).

#### 3.2.3. DLS Analysis

For dynamic light scattering (DLS), the size of the obtained silver nanoparticles was analyzed using the DLS method. For this purpose, Zetasizer Nano-S (Malvern Panalytical, Malvern, UK) was applied. Moreover, the zeta potential value of the synthesized AgNPs was registered and established at 0 mV. This value indicates that produced colloidal silver nanoparticles are unstable over time. This was also observed during experiments.

#### 3.2.4. Testing Microstructure

**Figure 7:**
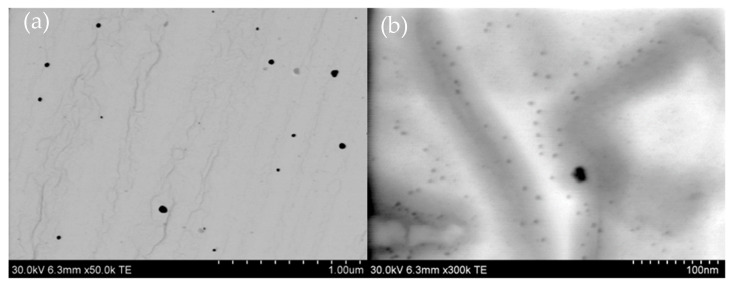
STEM analysis of 1 mM silver nanoparticles obtained via chemical reduction method using ascorbic acid (sample C1) at different magnifications of (**a**) 1 µm and (**b**) 100 nm. Initial conditions: 2 mM Ag(I), 20 mM AA, DMSO was used as a solvent, T = 22 °C.

The morphology of the silver nanoparticles was analyzed using the scanning transmission electron microscopy (STEM) mode of an ultra-high-resolution analytical Hitachi SU-70 Schottky field emission microscope (Hitachi High-Tech, Tokyo, Japan). Samples for STEM observations were deposited on fine copper grids and examined at an accelerating voltage of 30 kV across a wide range of magnifications. SEM analysis for the synthesized silver nanoparticles at the maximum concentration of 1 mM (sample C1) is depicted in Figure 7. Additionally, the morphology of AgNPs following the incorporation of cefiderocol was also examined, and the results are illustrated in Figure 7.

**Figure 8:**
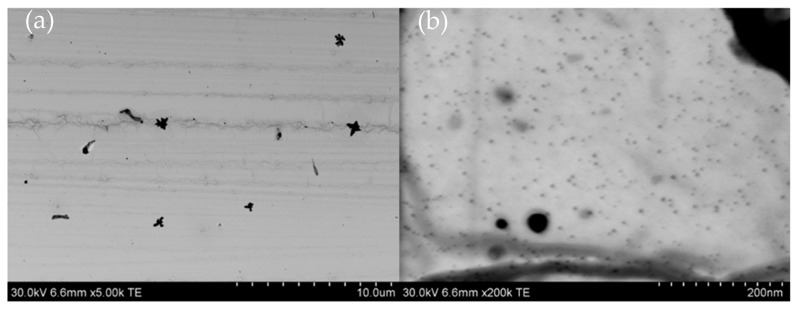
STEM analysis of silver nanoparticles with initial concentration of 1 mM mixed with CEF at different magnifications of (**a**) 10 µm and (**b**) 200 nm. Initial conditions: 1 mM AgNPs, 1.5 mg/mL CEF, DMSO was used as a solvent, T = 22 °C.

The obtained results confirmed that the chemical synthesis method produced silver nanoparticles with diameters ranging from 20 to 50 nm (Figure 7a), along with a substantial number of smaller spherical particles with diameters of approximately 5 nm visible at a higher magnification (Figure 7b). Following the addition of cefiderocol to the AgNPs, comparable results were observed (refer to Figure 8). It is worth noting that the addition of cefiderocol (CEF) to the colloidal silver induces a color change from yellow to yellow-gray, suggesting the possible appearance of aggregation or the formation of larger particles, even dendrites, depicted in Figure 8a.

Figure 8a indicates the coexistence of larger dendrite-like structures (Figure 8a) and much smaller spherical shape particle 20–40 nm, as well as numerous particles approximately 5 nm in size (Figure 8b).

### 3.3. Reagents

The following chemical reagents were used to synthesize silver nanoparticles:

*Dimethyl sulfoxide* (*DMSO*, Cat. No: 363550836, CAS: 67-68-5). In all experiments, DMSO (99%, for spectroscopy analysis, Thermo Scientific Chemicals, Waltham, MA, USA) was used as a solvent due to its ability to dissolve all components, i.e., AgNO_3_, ascorbic acid and cefiderocol (soluble in DMSO and only slightly soluble in water, according to https://www.medkoo.com/products/7445, accessed on 4 July 2025).

*Metal Precursor*. As a metal precursor, AgNO_3_ (Premion^®^, Thermo Scientific Chemicals, Waltham, MA, USA) 99.995% (metals basis min 63% Ag(I)) (Cat. No: 043087.14, CAS: 7761-88-8), powder was used. Before experiments, 10 mL of solution was prepared by dissolving 0.0034 g of salt in DMSO solution. The stock solution thus prepared has a concentration of 2 mM.

*Reductant*. As a silver ions reductant, L-ascorbic acid (AA, Warchem^®^, Zakręt, Poland, Cat. No: 41801, CAS: 50-81-7), was used. For this purpose, 0.0352 g of powder was dissolved in DMSO to obtain a final concentration of 20 mM.

### 3.4. Statistical Analysis

Because the dependent variables were not normally distributed, we applied generalized linear mixed modeling to obtain the relationship between inhibition zones and antibiotic concentrations, silver form, and its concentrations, as well as examining reference and MDR strains. We have chosen models based on the lowest Akaike’s information criterion [64] values and values of total deviance which were lower than 1.

## 4. Conclusions

Cefiderocol is effective against reference strains of *P. aeruginosa*, *E. coli*, and *A. baumannii*, showing dose-dependent inhibition. Multidrug-resistant (MDR) clinical strains of *A. baumannii* are resistant to both cefiderocol and various silver forms (ionic and nanoparticle). Silver nanoparticles (AgNPs) are generally more effective than ionic silver (Ag^+^) due to their unique physicochemical properties (small size, high surface activity, gradual ion release). The synergistic effects between cefiderocol and nanosilver are not consistent. For reference strains, the combination is less effective than cefiderocol alone, but for MDR and cefiderocol-resistant *A. baumannii*, the combination shows improved efficacy (mostly from 6 mm to 8 mm). High concentrations of silver can antagonize cefiderocol’s action by inhibiting bacterial surface proteins, including those involved in siderophore-mediated uptake, thus reducing cefiderocol’s effectiveness. Porins (e.g., OmpC, OmpF, OmpA) are susceptible to AgNPs, which can impair passive diffusion and nutrient transport, potentially affecting alternative entry routes for cefiderocol in resistant strains. If we want to produce wound dressings for external application, the vertical arrangement of both agents, cefiderocol and nanosilver, matters. Placing cefiderocol in direct contact with bacteria (as the lower layer) and nanosilver above (upper layer) leads to better antimicrobial outcomes. This setup allows cefiderocol to act directly on bacteria via its specific uptake mechanism, while nanosilver provides a sustained, broad-spectrum effect without interfering with cefiderocol’s transport. Optimal synergy is achieved at higher cefiderocol and lower nanosilver concentrations, especially in MDR strains (from average 14 mm at 1 mM of AgNPs to 17 mm at 0.5 mM of AgNPs for *A. baumannii* AB42 and from 9.3 mm at 1 mM of AgNPs to 13 mm at 0.5 mM of AgNPs for *A. baumannii* AB42). Our study successfully demonstrated the antimicrobial activity of cefiderocol and nanosilver compounds against Gram-negative bacteria, evidenced by the observed diameters of the inhibition zones. These initial findings provide a strong foundation for further investigation into their potential as therapeutic agents.

While minimum inhibitory concentrations (MICs) were not determined in this preliminary phase, we recognize their critical importance for a comprehensive understanding of antimicrobial potency. Therefore, our future studies will focus specifically on precisely ascertaining the MIC values for these compounds. This will include detailed investigations into the synergistic effects of nanosilver when combined with established antibiotics such as cefiderocol, utilizing methods like the checkerboard synergy assay. These subsequent studies will provide crucial insights into optimal dosing strategies and potential combination therapies, paving the way for their clinical applications.

## Figures and Tables

**Table 1:** Summary of the generalized linear mixed model with a gamma distribution and logarithmic link function analyzing silver concentration, silver form (nanosilver vs. Ag(I) ions), and bacterial strains tested at a cefiderocol concentration of 0.0075 mg/mL.

Effect	df	Wald’s Stat.	*p*
Intercept	1	99,378.27	0.00
Strain	4	3144.05	0.00
Silver	1	340.34	0.00
Ag conc	2	30.87	0.00
Strain × Silver	4	153.61	0.00
Strain × Ag conc	8	62.12	0.00
Silver × Ag conc	2	30.49	0.00
Strain × Silver × Ag conc	8	87.51	0.00

**Table 2:** Generalized linear mixed modeling of the inhibition zone diameter of examined strains in relation to cefiderocol concentration and its vertical orientation relative to nanosilver.

Effect	df	Wald’s Stat.	*p*
Intercept	1	193,111.7	0
CEF	4	4285.5	0
Position	2	2617.3	0
Strain	1	328.4	0
CEF × position	8	597	0
CEF × strain	4	70.5	0
Position × strain	2	7.5	0.02
CEF × position × strain	8	56.4	0

**Table 3:** Generalized linear mixed modeling of inhibition zone diameter of examined strains in relation to cefiderocol and nanosilver concentrations.

Variables	df	Wald’s Stat	*p*
*Pseudomonas aeruginosa* ATCC 27853			
Intercept	1	70,448.01	0.0000
Cef	1	4.49	0.0341
AgNPs	2	65.14	0.0000
Cef × AgNPs	2	10.1	0.0064
*Escherichia coli* ATCC 25922			
Intercept	1	148,964.9	0.0000
Cef	1	62.9	0.0000
AgNPs	2	105.8	0.0000
Cef × AgNPs	2	76.5	0.0000
*Acinetobacter baumannii* ATCC 19606			
Intercept	1	127,778.3	0.0000
Cef	1	507.1	0.0000
AgNPs	2	120.4	0.0000
Cef × AgNPs	2	73.7	0.0000
*Acinetobacter baumannii* AB 42			
Intercept	1	31,053.35	0.0000
Cef	1	234.25	0.0000
AgNPs	2	76.45	0.0000
Cef × AgNPs	2	16.1	0.0003
*Acinetobacter baumannii* AB 49			
Intercept	1	33,892.2	0.0000
Cef	1	227.16	0.0000
AgNPs	2	119.72	0.0000
Cef × AgNPs	2	35.68	0.0000

**Table 4:** Description of the preparation of solutions for testing.

Sample	Preparation	Ingredients in Sample (After Mixing)
BL_DMSO	DMSO	DMSO
BL_A	100 µL Ag(I) (2 mM) + 100 µL DMSO	AgNO_3_ concentration 1 mM
BL_B	No dilution	AgNPs concentration 1 mM
BL_C	100 µL CEF (1.5 mg/mL) + 100 µL DMSO	CEF concentration 0.75 mg/mL

Shortcut: BL—blank test, DMSO—dimethyl sulfoxide, A—Ag(I) silver ions, C—cefiderocol, B—AgNPs nanosilver particles.

**Table 5:** Material combinations used in the experiment—final sample.

Sample	Composition After Mixing: (100 µL A/B/C + 100 µL C1/C2/C3)
A_C1	CEF concentration 0.75 mg/mL + AgNPs (1 mM)
A_C2	CEF concentration 0.75 mg/mL + AgNPs (0.5 mM)
A_C3	CEF concentration 0.75 mg/mL + AgNPs (0.25 mM)
B_C1	CEF concentration 0.075 mg/mL + AgNPs (1 mM)
B_C2	CEF concentration 0.075 mg/mL + AgNPs (0.5 mM)
B_C3	CEF concentration 0.075 mg/mL + AgNPs (0.25 mM)
C_C1	CEF concentration 0.0075 mg/mL + AgNPs (1 mM)
C_C2	CEF concentration 0.0075 mg/mL + AgNPs (0.5 mM)
C_C3	CEF concentration 0.0075 mg/mL + AgNPs (0.25 mM)

Shortcut: A—CEF at initial concentration 1.5 mg/mL; B—CEF at initial concentration 0.15 mg/mL; C—CEF at initial concentration 0.015 mg/mL, C1—AgNPs at initial concentration 1 mM; C2—AgNPs at initial concentration 0.5 mM; C3—AgNPs at initial concentration 0.25 mM. AgNPs (C1) taken 10 min after mixing Ag(I) ions with the reducer, yellow color.

## Data Availability

Data will be made available on request.
